# Leveraging women empowerment for better maternal health: using the SWPER index to identify key drivers of quality antenatal care in Southern Africa

**DOI:** 10.3389/fgwh.2026.1886495

**Published:** 2026-10-02

**Authors:** Munawar Harun Koray, Evelina Ndapewa Johannes, Emmanuel Kwasi Afriyie, Alfred Pie Faabie

**Affiliations:** 1 Upper West Regional Health Directorate, Ghana Health Service. Wa, Ghana; 2 Southern Medical University, Guangzhou, China; 3 Komfo Anokye Teaching Hospital, Kumasi, Ghana

**Keywords:** antenatal care, maternal health, multilevel analysis, Southern Africa, SWPER index, women's empowerment

## Abstract

**Background:**

Quality antenatal care (ANC) is essential for reducing maternal and neonatal mortality; however, access remains inequitable in Southern Africa. Women's empowerment is increasingly recognized as a key determinant of health-seeking behavior; however, its role in shaping ANC quality has not been adequately examined in the region.

**Methods:**

This study used cross-sectional Demographic and Health Survey (DHS) data from 2015 to 2023 across seven Southern African countries (*n* = 36,671). Women's empowerment was measured using the SWPER Global Index, which covers the domains of attitude toward violence, social independence, and decision-making. Quality ANC was defined as the receipt of four essential services: blood pressure measurement, urine testing, blood testing, and iron supplementation. Hierarchical models were fitted, and the measures of variation and model fit were evaluated. The significance level was set at *p* < 0.05, with a 95% confidence interval.

**Results:**

Overall, 45.1% of the women received quality ANC, with wide variation between countries (27.4% in Malawi vs. 92.1% in South Africa). A high level of empowerment against violence and decision-making was observed among the women in most of the countries. Women with high social independence (aOR = 1.12, 95% CI: 1.01–1.24) and strong rejection of gender-based violence (aOR = 1.15, 95% CI: 1.05–1.26) were significantly more likely to receive high-quality ANC. Early ANC initiation, ≥8 visits, and doctor-led care were the most powerful predictors of quality ANC care.

**Conclusion:**

Women's empowerment, particularly social independence and autonomy from violence, plays a critical role in improving the quality of ANC in Southern Africa. However, persistent socioeconomic and geographic inequities highlight the need for context-specific strategies. Policies that integrate gender equality frameworks with strengthened ANC service delivery, especially in rural and low-performing countries, are essential for achieving maternal health targets.

## Introduction

Quality antenatal care (ANC) is fundamental for safeguarding maternal and neonatal health. Maternal health is recognized as a significant public health challenge, with a burden disproportionately affecting women in Southern Africa. Although diversity and divergence are defining characteristics of poor maternal health in the 21st century, strong political, research, and technical commitment to improving equity-sensitive information systems is required to monitor the gap in maternal health (1). Maternal deaths are often solitary and hidden events that go uncounted due to various factors (2). The World Health Organization (WHO) reports that in 2023, over 700 women die daily from preventable causes associated with pregnancy and delivery, with nearly 92% of all maternal deaths occurring in low- and lower-middle-income countries (3). From 2000 to 2020, only 52% of births involved a skilled birth attendant in the 81 countries, while 54% of pregnant individuals had ≥4 antenatal visits (3).

Antenatal care (ANC) is care provided to pregnant women and teenage girls by professional health care providers to ensure the best health outcomes for mothers and babies during pregnancy. The 2016 WHO ANC model emphasizes that quality antenatal care is essential for improving maternal outcomes, an issue of particular importance in Southern Africa (4, 5). In low- and middle-income countries, many pregnant women register for ANC but do not continue the care continuum to institutional deliveries. Most inequities in access to antenatal care are driven by differences in wealth, ethnicity, geography, and the availability of health infrastructure (6).

Compared to Southern Africa, evidence from Nigeria, Chad, Liberia, and Sierra Leone shows that women from socioeconomically disadvantaged households have lower ANC access and higher mortality/morbidity (7, 8). Reports have shown that early adolescent childbearing increases risks for mothers and infants and is associated with lower education and poverty. Sub-Saharan Africa (SSA), Central, and Southern Asia remain the largest contributors to the maternal mortality burden. Despite a 40% decline in maternal mortality, from 727 to 442 deaths per 100,000 live births between 2000 and 2023, the region still accounts for 70% of global maternaldeaths (3, 9)*.* A micro-level analysis In South Africa found that 80% of maternal deaths occurred despite ANC but with substandard care in district hospitals and a lack of emergency and specialist care (10). The current pace of progress in preventing maternal deaths in SSA is significantly less than the pace of progress required to bring the region closer to the Sustainable Development Goals (SDGs) target of 70 maternal deaths per 100,000 births by 2030 (11, 12).

Although the coverage of maternal health services has increased in Namibia and Kenya, lengthy wait times still affect patient satisfaction. The technical competence of providers may not be sufficient to deliver high-quality care (13). Across six sub-Saharan African countries, facilities with up-to-date guidelines, proper equipment, electricity, and water were significantly more likely to provide high-quality care (14). However, cultural beliefs, age, marital status, mothers education, partners' formal education, negative attitudes towards health providers, long distance to and from ANC health care facilities, fear of HIV testing and results, inability to determine pregnancy at earlier stages, and financial constraints also remain significant contributors to maternal mortality rates and preventable complications in southern Africa (14–16). Therefore, ample recognition should be accorded to the existing and potential facilitators and barriers to quality ANC across and within countries (17).

The concept of women's empowerment is multifaceted and includes social, economic, and decision-making power, all of which impact how people seek healthcare. Research has demonstrated the importance of empowerment for health outcomes in Sub-Saharan Africa by measuring it using metrics such as freedom of mobility, autonomy in household decision-making, and rejection of gender-based violence (18). Although the impacts differ depending vary across environments, Bayesian multilevel models also show that gender relations and empowerment are significant predictors of maternal health outcomes. Systematic reviews have also confirmed that maternal and child health outcomes in Sub-Saharan Africa are significantly impacted by empowerment through agency, autonomy, and control over resources (19, 20).

Research consistently shows that empowered women are more likely to access and utilize essential maternal health services such as antenatal care, skilled birth attendance, and postnatal care. A meta-analysis of 33 countries in sub-Saharan Africa found that empowered women had significantly higher odds of attending antenatal care than less-empowered peers. Similarly, empowerment indicators, such as decision-making autonomy and disapproval of wife beating, were linked to improved quality of antenatal care in Cameroon. Evidence from Ethiopia shows that women with greater decision-making power are more likely to deliver in health facilities, reinforcing the role of empowerment in safe childbirth practices (20–22).

Despite these findings, research specifically linking empowerment to the quality of ANC in Southern Africa remains limited, with most studies focusing on coverage, number of visits, or socioeconomic inequalities rather than the content and quality of maternal care. There is also limited research using the SWPER Index to explore empowerment dimensions beyond education and wealth for quality antenatal care. Therefore, this study uses nationally representative datasets from various Demographic and Health Surveys (DHSs) to investigate the effect of women's empowerment on the quality of ANC care across seven Southern African countries.

### Study objectives

1. To examine the prevalence of quality ANC in Southern Africa
2. To assess the level of empowerment among women living in Southern Africa
3. To ascertain the effect of women's empowerment on the quality of ANC.

### Conceptual framework

The conceptual framework for this study (Supplementary Figure S1) outlines a comprehensive model for investigating the effect of women's empowerment on ANC quality. It is grounded in Kabeer's (23) resources–agency–achievements framework of women's empowerment (23). The three SWPER Global domains used in this study represent measurable dimensions of “agency”: a woman's capacity to define and act on her own goals, including those related to her own health and that of her children. At its core, the framework posits that women's empowerment, defined by their attitudes toward violence, social independence, and decision-making power, is a fundamental factor influencing their ability to seek, access, and receive quality ANC services. This central relationship suggests that more empowered women are better equipped to prioritize their health, navigate the healthcare system, and demand higher standards of care.

However, the framework importantly recognizes that this relationship is not simple or isolated. It is shaped and mediated by various individual and community-level factors. These are described as resources under Kabeer's framework (23). A woman's sociodemographic characteristics, such as her age, religion, education, occupation, and wealth, form a foundational layer that could influence both her level of empowerment and her health-seeking behavior. Furthermore, exposure to media through radio, television, and newspapers was identified as a key factor that can increase health awareness and amplify the effect of empowerment. Specific health service factors related to the current pregnancy, including the timing of ANC registration, the number of visits, and the type of provider, were also included as variables that could directly affect the quality metric.

Finally, the framework places this entire individual-level interplay within the broader contextual environment of community and household factors. Conditions such as the sex of the household head, distance to a health facility, place of residence (urban or rural), and country are critical external factors that can either enable or constrain a woman's agency and access to care, regardless of her personal empowerment level. In essence, this model provides a multi-layered analysis, arguing that the quality of ANC a woman receives is the result of a complex interaction between her personal empowerment, individual circumstances, and the structural environment in which she lives.

## Methods

### Study design and data source

This study utilized a cross-sectional approach, drawing on existing secondary data from the Demographic and Health Survey (DHS) of seven Southern African countries. Demographic and Health Surveys (DHSs) are cross-sectional surveys that are nationally representative and are conducted in various low- and middle-income nations. These surveys usually include 5,000–30,000 households (24). The data for this study cover the years 2015–2023 and were collected using a standardized questionnaire model, although it can be modified and optional modules may be added. Typically, the data are derived from women's self-reports. Information on ANC is gathered for the most recent pregnancy that led to a live birth within a specified recall period, generally within the past five years. The complete methodology used in the DHS is presented on the website and in the literature (https://www.dhsprogram.com/Methodology/Survey-Types/DHS-Methodology.cfm) and in a previous study (24).

### Study population and sample

Respondents to these surveys are recruited using a multistage random sampling method, and samples are generally representative at the national, residential (urban-rural), and regional (counties, cities, etc.) levels. The population for this study included 109,888 women aged 15–49 years in the countries included in the study (Supplementary Table S1). The study included women who were either married or cohabiting with their partners and had registered and visited ANC at least once. This inclusion criterion reduced our sample size to 36,671 (weighted sample = 35,862).

### Study variables

#### Outcome variable

The primary outcome variable for this study is “Quality ANC.” Quality ANC is characterized by the percentage of mothers who received four key services: (1) blood pressure measurement, (2) urine sample collection, (3) administration of iron supplements, and (4) blood sample collection during any ANC visit. This definition was derived from a study by Field (17). Women who received all four services were classified as having received quality ANC (coded as 1), while those who did not receive all four services were categorised otherwise (coded as 0). To qualify for quality ANC, a woman must receive at least these four services. While the WHO advises that women should receive more than just these four services for optimal ANC (5), this study concentrated on these four due to the constraints of variable availability, comparability, and the specific DHS survey wave across the SSA. Throughout this manuscript, “quality ANC” refers specifically to receipt of these four structural components and should be read as a proxy indicator rather than a comprehensive assessment of ANC quality, which would also encompass interpersonal, informational, and content dimensions of care not captured in the DHS.

#### Predictor variables

The primary predictor variable in this study was the degree of women's empowerment, as measured by the SWPER Global index (25, 26). The SWPER Global women's empowerment index was formulated using data from the Demographic and Health Survey (DHS) conducted across 62 low- and middle-income countries (25, 26). The SWPER Global index comprises three fundamental domains: i) the attitude towards violence domain, ii) the social independence domain, and iii) the decision-making domain, derived from 14 questions in the DHS women's questionnaire. According to the SWPER Global methodology, women are classified into three levels of empowerment: low, medium, and high empowerment. The development and validation of this empowerment index have been thoroughly documented by Ewerling et al. (25, 26).

The attitude-to-violence domain is derived from 5 items (whether wife-beating is justified if she burns food, argues, goes out without telling her husband, neglects the children, or refuses sex); the social-independence domain from 6 items (education level, education gap with partner, age gap with partner, age at first cohabitation, age at first birth, and ideal number of children); and the decision-making domain from 3 items (who usually decides on the respondent's own health care, on large household purchases, and on visits to family or relatives). Classification into low/medium/high empowerment groups followed the standard three-group cut-points generated by the official SWPER Global STATA syntax available at https://goo.gl/isGonn (26).

The attitude towards violence domain assesses women's perceptions regarding the justification of wife beating under specific circumstances. Attitude towards violence: the high category represents high disagreement or rejection of an attitude towards violence (positive), with the low category emphasizing high acceptance of violence (negative).

The social independence domain evaluates women's information access, educational achievement, marital age, age at first birth, and spousal age and education. The decision-making domain examines household decision-making dynamics and women's employment status.

This study also incorporated covariates that have been identified as significant predictors of ANC in previous research (17, 27). These covariates were grouped into demographic characteristics (age, religion, educational level, wealth index, and maternal occupation), health service factors (gestational age at ANC registration, number of ANC visits, and person providing ANC services), exposure to media (frequency of listening to radio, frequency of watching television, and frequency of reading newspaper/magazine), and community-level factors (place of residence, region, and distance to health facility).

### Data analysis

This study utilized a thorough three-step analytical approach: (1) descriptive analysis, (2) bivariate analysis, and (3) multilevel logistic regression. Each step was methodically constructed on the foundation of the previous step to deliver a detailed understanding of quality ANC. During the descriptive analysis, we evaluated the prevalence of quality ANC across all seven countries involved in the study, employing a forest plot, as well as across all covariates. Additionally, the distribution of women's empowerment levels was examined across all three domains.

Pearson's chi-square test was utilized in the bivariate analysis to investigate the connection between women's empowerment and the quality of ANC, along with the covariates. The significance level for this analysis was established at *p* ≤ 0.05.

In the third stage, a multilevel analysis was performed to address the hierarchical structure of the DHS survey. Variables that were statistically significant with a *p*-value of ≤0.05 were incorporated into the regression models. Four different models were developed to determine which one best represented the data. The initial model (Model I, or the null model) did not include any explanatory variables and was used to estimate the random variation in the intercept and the intra-cluster correlation (ICC) coefficient, aiming to assess the degree of cluster variation in quality ANC. The second model (Model II) examined the relationship between quality ANC and individual-level variables, namely demographic characteristics, ANC service factors, and media exposure (including the SWPER empowerment domains). The third model (Model III) explored the impact of community-level factors (place of residence, distance to health facility, sex of household head, and country) on quality ANC. The final model (Model IV) combined both individual and community-level factors, controlling for all variables to pinpoint the true predictors of quality ANC. Statistical significance of the predictor variables was determined using a *p*-value of <0.05 and an adjusted odds ratio (aOR) with a 95% confidence interval.

The study employed intra-class correlation (ICC), proportion change in variance (PCV), and median odds ratio (MOR) to assess the prevalence of quality ANC across clusters. The MOR is defined as the median value of the odds ratio between the area at the highest risk and the area at the lowest risk of quality ANC when randomly picking out two areas. It measures the unexplained cluster heterogeneity when randomly picking out two clusters (28).

The MOR is calculated using this formula;

$$\mathrm{MOR}=\exp.\left[\sqrt{(2\times \mathrm{VA})\times 0.6745}\right],$$

where VA is the area level variance (29).

The proportional change in variance is calculated using this formula;

[PCV=(VA−VB)/VA)×100]

where VA, Variance of initial model and VB,Variance of model with more terms measures the total variation attributed by individual level and community level factors in the multilevel model (30).

The data was analysed using STATA and the figures and tables were created using Microsoft Excel. In the analysis, the “svy” command in STATA was used to allow for adjustments for the cluster-sampling design of the data collection and weights. This was also done to account for selection bias in the study. The “melogit” STATA command was also used for the logistic regression, “estat icc” for the intra-class correlations and “estat ic” for the information criterions (Akaike Information Criterion (AIC) and Bayesian Information Criterion (BIC).

### Missing data

STATA's melogit command uses listwise (complete-case) deletion by default. Observations missing a value on any variable included in a given bivariate or multilevel model were excluded from that specific analysis. This accounts for the smaller analytic sample used in the bivariate and multilevel analyses (*n* = 32,880, weighted) relative to the overall eligible sample described above (*n* = 35,862, weighted). This reduction is attributable to missing values on one or more covariates (e.g., wealth index, media exposure, or ANC service variables). The number of women excluded due to missingness on each key covariate is summarised in Supplementary Table S2.

### Model diagnostics

Given the complexity inherent in the study's sampling design, the hierarchical structure of the fitted model, and the categorical nature of the outcome variable, the model's collinearity was evaluated using the generalized variance inflation factor (GVIF). To obtain the GVIF of the independent variables, the variance inflation factor was first obtained from the post-estimation results (31). The GVIF was then computed using:

$$\mathrm{GVIF}=\mathrm{VI}F^{\left[1/(2*\mathrm{df})\right]}$$

where df, degree of freedom = 19.

Corresponding to the 19 categorical predictor variables entered into the full model, as listed in Supplementary Table S1. Each variable's individual GVIF^(1/2df) value was computed from its own degrees of freedom (number of categories—1), and the reported overall GVIF is 1.31, representing a model-level summary calculated as the mean of the 19 individual GVIF^(1/2df) values, rather than a single-variable measure, indicating that the overall variance of the model coefficients is inflated by 31%, consistent with the absence of problematic multicollinearity in the model (Supplementary Table S2).

## Results

Figure 1 shows a significant difference (*χ*^2^ = 592.84, *p* < 0.001) in the quality of ANC between the countries. A 92.1% (CI = 89.7%—93.9%) and 87.4% (CI = 84.5%—89.8%) of women in South Africa and Lesotho, respectively, received quality ANC. On the other hand, 27.4% (CI = 26.4%—28.5%) and 39.8% (CI = 38.1%—41.5%) of women received quality ANC in Malawi and Congo DR, respectively. Overall, less than half [45.1% (CI = 37.0%—53.2%)] of women included in the study received quality ANC.

**Figure 1:**
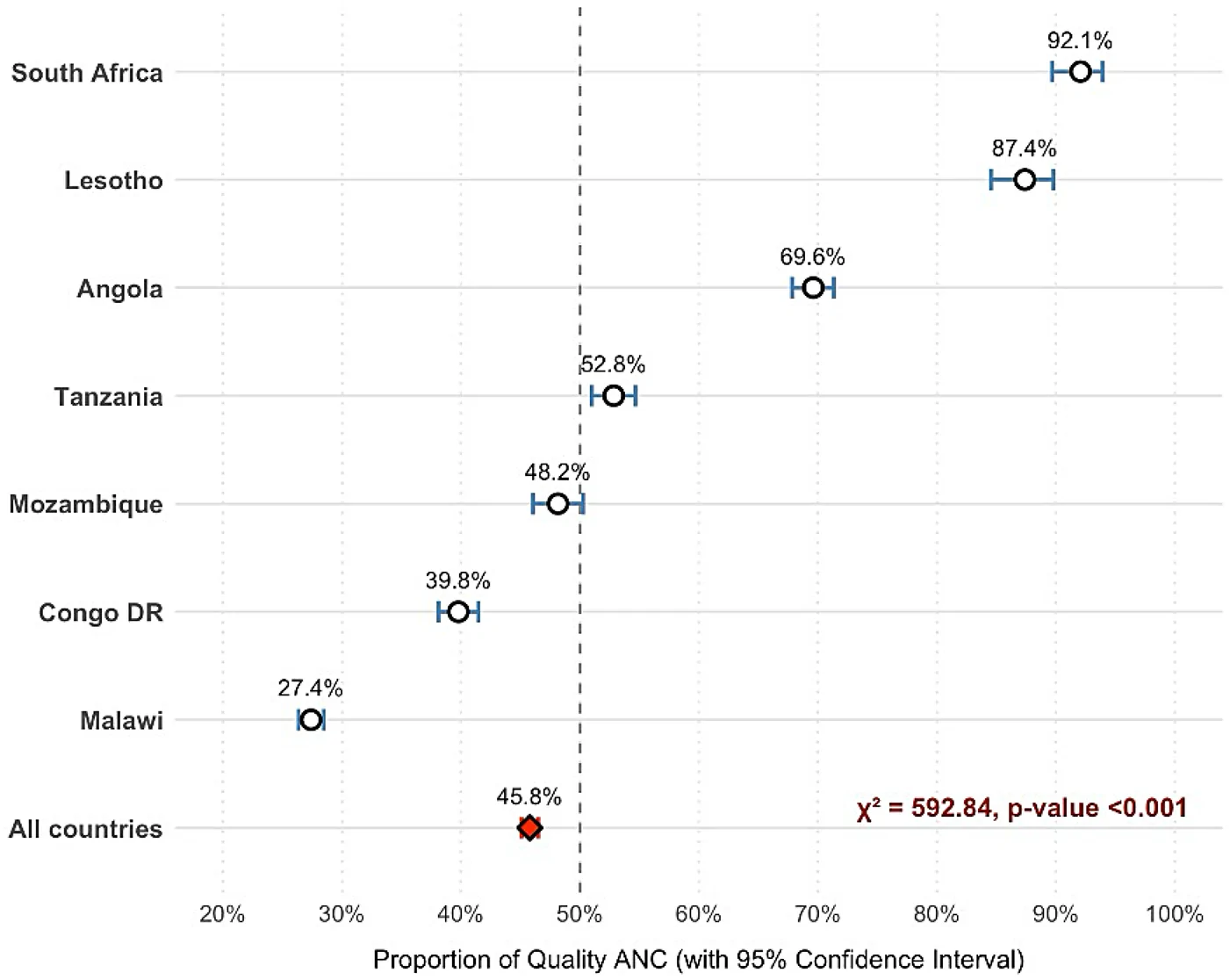
Proportion of quality ANC across countries.

**Figure 2:**
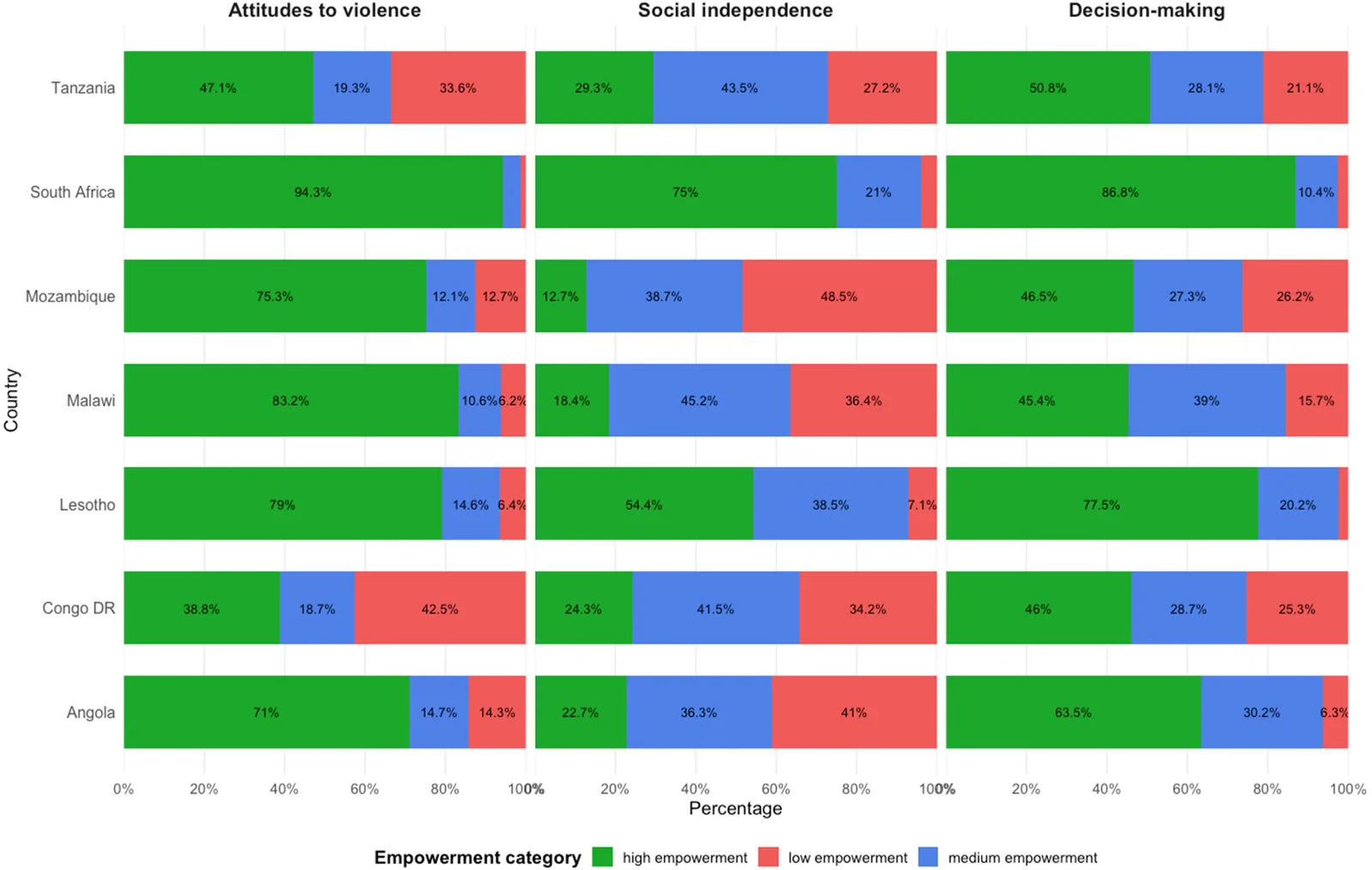
Women empowerment across countries in the study.

Figure 2 reveals significant variations (*χ*^2^ = 6,065.52, *p* < 0.0001) in women empowerment attitude to violence. Most of the countries showed high level of empowerment, with South Africa reaching 94.3%. This implies majority of women in these countries reject attitudes that justify gender-based violence. Conversely, countries like Tanzania (47.1%) and Congo DR (38.8%) exhibited lower levels of empowerment.

Social independence among the women differed substantially (*χ*^2^ = 3,020.34, *p* < 0.0001) across the countries. South Africa (75%) and Lesotho (54.4%) shows the highest levels of empowerment in this domain. Countries such as Tanzania (43.5%), and Malawi (45.2%) presented moderate levels of empowerment in social independence. Most of the women in Mozambique (48.5%) and Angola (36.3%) had low levels of empowerment in this domain.

A significant difference (*χ*^2^ = 2,145.92, *p* < 0.0001) is also observed in the decision making domain across the countries, although the majority of women in all the countries exhibited high empowerment levels. South Africa (86.8%), and Lesotho (77.5%) presented the highest levels of high empowerment. Tanzania (50.8%), Malawi (45.4%), Congo DR (46.0%), and Mozambique (46.5%) reflect more balanced but lower levels of empowerment, with higher shares of low and medium empowerment.

There was a clear and consistent gradient between women's empowerment and utilization of quality antenatal care (ANC) services across all three SWPER domains. In the attitude to violence domain, the proportion of women receiving quality ANC increased from 32.0% among those with low empowerment to 39.1% among those with medium empowerment and 45.8% among those with high empowerment. The association between attitude toward violence and receipt of quality ANC was statistically significant (*χ*^2^ = 79.16, *p* < 0.0001).

Similarly, a pronounced gradient was observed for social independence. Quality ANC utilization increased from 32.5% among women with low social independence to 39.8% among those with medium social independence and 59.4% among those with high social independence. This association was statistically significant (*χ*^2^ = 335.93, *p* < 0.0001).

For decision-making, quality ANC utilization also increased with increasing empowerment, from 29.4% among women with low decision-making empowerment to 38.0% among those with medium empowerment and 48.7% among those with high empowerment. The association was statistically significant (*χ*^2^ = 162.62, *p* < 0.0001). This is shown in Table 1.

**Table 1:** Prevalence of quality ANC and its association (chi-square test) with the women empowerment domains.

SWPER Domains	Quality ANC (Weighted)		Chi-square	*p*-value
	No (%)	Yes (%)		
Attitude to violence			79.16	<0.0001
Low empowerment	4,989 (68.0)	2,351 (32.0)		
Medium empowerment	3,151 (61.0)	2,019 (39.1)		
High empowerment	13,000 (54.2)	11,000 (45.8)		
Social independence			335.93	<0.0001
Low empowerment	8,409 (67.5)	4,055 (32.5)		
Medium empowerment	8,859 (60.3)	5,846 (39.8)		
High empowerment	3,534 (40.7)	5,159 (59.4)		
Decision-making			162.62	<0.0001
Low empowerment	4,453 (70.6)	1,855 (29.4)		
Medium empowerment	6,884 (62.0)	4,218 (38.0)		
High empowerment	9,465 (51.3)	8,987 (48.7)		

### Demographic characteristics

The majority of women are between the ages of 20 and 34 (70.7%), with the largest group being 20–24 years old (26.2%). Christianity is the predominant religion (84.8%). In terms of education, nearly half have primary education (46.1%), while only 3.6% have higher education. The wealth distribution is relatively balanced across quintiles, though slightly more women are in the poorer and poorest categories. The population is predominantly rural (68.3%). Media exposure is low overall, particularly for reading newspapers (83.4% not at all) and watching television (67.9% not at all). For maternal health, most women (52.1%) had 4–7 ANC visits, and over half registered for ANC between 4 and 6 months of pregnancy (55.1%). The primary assistance during ANC was from a nurse or midwife (60.2%). About half (54.3%) reported that distance to a health facility was not a big problem, and the vast majority of households were headed by males (83.7%). Malawi contribute the most of the study sample (30.3%), while Lesotho contributes the least (2.7%). This is presented in Supplementary Table S3.

### Association between demographic factors and quality antenatal care

Socio-demographic factors were significantly associated with the uptake of quality antenatal care as presented in Table 2. Maternal education exhibited a pronounced variation, with 27.7% of women lacking formal education receiving quality antenatal care, in contrast to 84.7% of those with higher education (*χ*^2^ = 2,618.99, *p* < 0.0001). Age demonstrated a non-linear relationship with uptake, reaching a peak of 45.7% among women aged 40–44 years and declining to 33.4% in the 45–49 age group (*χ*^2^ = 179.39, *p* < 0.0001).

**Table 2:** Association between demographic factors and quality antenatal.

Socio-demographic Characteristics	Quality Antenatal Care		*χ* ^2^	*p*-value
	No (%)	Yes (%)		
Age group of women			**179.39**	**<0** **.** **0001**
15–19	1,651 (62.9)	974 (37.1)		
20–24	5,839 (62.0)	3,572 (38.0)		
25–29	4,828 (54.6)	4,013 (45.4)		
30–34	4,010 (56.6)	3,076 (43.4)		
35–39	2,781 (56.4)	2,152 (43.6)		
40–44	1,247 (54.3)	1,051 (45.7)		
45–49	445 (66.6)	223 (33.4)		
Religion			**116** **.** **41**	**<0** **.** **0001**
Christianity	15,526 (60.8)	10,007 (39.2)		
Islam	1,772 (69.7)	769 (30.3)		
Animist/Traditional	385 (65.5)	203 (34.5)		
No religion	772 (54.3)	650 (45.7)		
Other	25 (57.3)	19 (42.7)		
Educational status			**2,618.99**	**<0** **.** **0001**
no education	4,668 (72.3)	1,787 (27.7)		
primary	10,677 (64.6)	5,846 (35.4)		
secondary	5,262 (45.3)	6,345 (54.7)		
higher	195 (15.3)	1,082 (84.7)		
Wealth index			**3,276.10**	**<0** **.** **0001**
poorest	5,766 (73.9)	2,039 (26.1)		
poorer	5,502 (69.7)	2,387 (30.3)		
middle	4,218 (60.0)	2,818 (40.0)		
richer	3,246 (47.4)	3,598 (52.6)		
richest	2,070 (32.9)	4,219 (67.1)		
Place of residence			**4,191.05**	**<0** **.** **0001**
urban	3,802 (33.5)	7,551 (66.5)		
rural	17,000 (69.4)	7,510 (30.6)		
Maternal Occupation			**2,779.80**	**<0** **.** **0001**
not working	6,837 (53.4)	5,966 (46.6)		
Professional/Technical	601 (36.5)	1,045 (63.5)		
Sales	907 (35.3)	1,663 (64.7)		
Agricultural	9,148 (74.6)	3,119 (25.4)		
Household/Domestic	1,185 (44.8)	1,460 (55.2)		
Other services	63 (25.3)	186 (74.7)		
Manual worker	2,009 (57.5)	1,488 (42.5)		
Other/Missing	51 (27.9)	133 (72.1)		
Frequency of reading newspaper			**1,061.86**	**<0.0001**
not at all	18,460 (61.7)	11,457 (38.3)		
at least once a week	1,550 (41.9)	2,153 (58.1)		
almost every day	791 (35.3)	1,451 (64.7)		
Frequency of listening to radio			**951.07**	**<0.0001**
not at all	12,736 (65.2)	6,798 (34.8)		
at least once a week	3,553 (51.3)	3,368 (48.7)		
almost every day	4,513 (48.0)	4,894 (52.0)		
Frequency of watching television			**4,419.86**	**<0.0001**
not at all	16,858 (69.2)	7,503 (30.8)		
at least once a week	1,773 (47.9)	1,932 (52.1)		
almost every day	2,170 (27.8)	5,625 (72.2)		
Number of ANC visits			**4,244.29**	**<0** **.** **0001**
No ANC	2,982 (100.0)	0 (0.0)		
Below 4	8,392 (67.5)	4,036 (32.5)		
4–7 visits	8,939 (48.0)	9,697 (52.0)		
8 or more	443 (25.4)	1,299 (74.6)		
Time of ANC registration			**3,395.28**	**<0.0001**
No ANC	2,982 (100.0)	0 (0.0)		
1–3 months	4,436 (42.2)	6,068 (57.8)		
4–6 months	11,605 (58.9)	8,105 (41.1)		
7 + months	1,733 (66.8)	860 (33.2)		
Person providing assistance during ANC			**2,958.90**	**<0.0001**
Doctor	2,644 (45.9)	3,123 (54.1)		
Nurse/Midwife	12,488 (58.3)	8,939 (41.7)		
Other health worker	2,111 (43.8)	2,706 (56.2)		
TBA/Other	418 (67.2)	204 (32.8)		
No ANC	2,939 (100.0)	0 (0.0)		
Distance to health facility a problem			**576.51**	**<0.0001**
not a big problem	10,163 (53.2)	8,943 (46.8)		
big problem	10,576 (65.7)	5,527 (34.3)		
Sex of household head			**37.02**	**<0** **.** **0001**
male	17,619 (58.7)	12,398 (41.3)		
female	3,183 (54.5)	2,662 (45.5)		

**Bold value indicates Variables significant at** ***p*** **< 0.05**

Economic factors revealed substantial disparities in quality ANC access. A stark wealth gradient was evident, with uptake ranging from 26.1% among women in the poorest wealth quintile to 67.1% among those in the richest quintile (*χ*^2^ = 3,276.10, *p* < 0.0001). Occupational status further reinforced this economic divide, as women in agricultural work had the lowest uptake (25.4%) while those in professional and technical occupations reached 63.5% uptake (*χ*^2^ = 2,779.80, *p* < 0.0001).

Geographic and household factors showed pronounced disparities in care quality. Urban women were more than twice as likely to receive quality ANC (66.5%) compared to their rural counterparts (30.6%) (*χ*^2^ = 4,191.05, *p* < 0.0001). Media exposure emerged as a crucial factor in quality ANC utilization. Television access showed the strongest effect, with daily viewers achieving 72.2% uptake compared to 30.8% among non-viewers (*χ*^2^ = 4,419.86, *p* < 0.0001). Regular newspaper readers also demonstrated higher uptake (58.1%) than those who never read newspapers (38.3%) (*χ*^2^ = 1,061.86, *p* < 0.0001). Healthcare service factors were critical determinants of care quality. The number of ANC visits showed a strong relationship, with women attending 8 or more visits achieving 74.6% uptake compared to only 32.5% among those with fewer than 4 visits (*χ*^2^ = 4,244.29, *p* < 0.0001). Early initiation of ANC care was equally important, as first-trimester registration resulted in 57.8% uptake vs. 33.2% for registration after 7 months (*χ*^2^ = 3,395.28, *p* < 0.0001).

### Multilevel logistic regression

After adjusting for all socio-demographic, media exposure, ANC service characteristics, and health system factors (Model IV), two of the three SWPER domains remained significantly associated with higher odds of receiving quality ANC.

Women with high social independence had 1.12 times higher odds of receiving quality ANC compared to women with low social independence (aOR = 1.117, 95% CI: 1.007–1.240, *p* < 0.05). A positive, though slightly attenuated, association was also observed for medium social independence (aOR = 1.110, 95% CI: 1.032–1.194, *p* < 0.01). The domain measuring attitude to violence also showed a significant relationship. Women with high empowerment in this domain, indicating a lower tolerance for domestic violence, had 1.15 times higher odds of receiving quality ANC (aOR = 1.148, 95% CI: 1.045–1.261, *p* < 0.01). The association for medium empowerment was not statistically significant in the full model. The decision-making domain presented a complex picture. While high empowerment was not statistically significant in the full model, medium empowerment was associated with 11% lower odds of receiving quality ANC compared to low empowerment (aOR = 0.893, 95% CI: 0.812–0.981, *p* < 0.05).

Wealth and education demonstrated strong, graded relationships with the outcome. Women in the richest wealth quintile had 1.7 times higher odds of receiving quality ANC than those in the poorest quintile (aOR = 1.705, 95% CI: 1.466–1.982, *p* < 0.001). Similarly, women with higher education had 1.9 times higher odds than those with no education (aOR = 1.879, 95% CI: 1.442–2.447, *p* < 0.001).

Maternal occupation was another significant factor. Women working in agricultural occupations had consistently lower odds of receiving quality ANC across all models (aOR = 0.826, 95% CI: 0.760–0.897, *p* < 0.001). Notably, women aged 40–44 had significantly higher odds of receiving quality ANC compared to adolescents (15–19 years) (aOR = 1.311, 95% CI: 1.110–1.550, *p* < 0.01). Media exposure, particularly through television, was positively associated with quality ANC. Women who watched television almost every day had 1.3 times higher odds than those who did not watch at all (aOR = 1.319, 95% CI: 1.179–1.475, *p* < 0.001). Exposure to newspapers and radio was not significantly associated.

Service-related factors were among the strongest predictors of quality ANC. As expected, a higher number of ANC visits was strongly associated with increased odds. Women with 8 or more visits had 1.5 times higher odds than those with fewer than 4 visits (aOR = 1.456, 95% CI: 1.230–1.724, *p* < 0.001). Early initiation of ANC was also crucial. Registering for ANC at 7 months or later was associated with a 38% reduction in the odds of receiving quality care compared to registering in the first trimester (aOR = 0.621, 95% CI: 0.542–0.712, *p* < 0.001). The type of provider was significantly associated with quality. Compared to doctors, receiving care from a nurse/midwife (aOR = 0.773), other health worker (aOR = 0.619), or a traditional birth attendant/other (aOR = 0.584) was associated with lower odds of receiving quality ANC (all *p* < 0.001).

Significant disparities were observed based on geographical location and access. Women living in rural areas had 33% lower odds of receiving quality ANC than their urban counterparts (aOR = 0.675, 95% CI: 0.593–0.768, *p* < 0.001). Perceiving distance to a health facility as a big problem was not statistically significant in the full model. There were profound country-level variations. Using South Africa as the reference, women in Lesotho had dramatically higher odds of receiving quality ANC (aOR = 7.193, 95% CI: 5.432–9.525, *p* < 0.001). Conversely, women in Congo DR (aOR = 0.457) and Malawi (aOR = 0.343) had significantly lower odds (*p* < 0.001). Women in Angola had slightly higher odds than those in South Africa (aOR = 1.327, *p* < 0.01). This is presented in Table 3.

**Table 3:** Multi-level logistic regression models.

Variable	Model I Empty model	Model II aOR (95% CI) (Individual-level)	Model III aOR (95% CI) (Community-level)	Model IV aOR (95% CI) (Full model)
SWPER Domains				
Attitude to violence				
Low empowerment		Ref		Ref
Medium empowerment		1.164 (1.045–1.296)**		1.074 (0.963–1.197)
High empowerment		1.230 (1.123–1.346)***		1.148 (1.045–1.261)**
Social independence				
Low empowerment		Ref		Ref
Medium empowerment		1.121 (1.042–1.205)**		1.110 (1.032–1.194)**
High empowerment		1.165 (1.051–1.292)**		1.117 (1.007–1.240)*
Decision-making				
Low empowerment		Ref		Ref
Medium empowerment		0.963 (0.876–1.058)		0.893 (0.812–0.981)*
High empowerment		1.244 (1.136–1.363)***		1.087 (0.993–1.191)
Socio-demographic Characteristics				
Age group of women				
15–19		Ref		Ref
20–24		0.925 (0.820–1.042)		0.954 (0.846–1.077)
25–29		1.002 (0.886–1.134)		1.047 (0.925–1.186)
30–34		1.020 (0.896–1.161)		1.067 (0.937–1.216)
35–39		1.057 (0.920–1.214)		1.118 (0.972–1.285)
40–44		1.274 (1.079–1.504)**		1.311 (1.110–1.550)**
45–49		0.924 (0.715–1.194)		0.979 (0.757–1.266)
Religion				
Christianity		Ref		Ref
Islam		0.853 (0.739–0.985)*		0.961 (0.831–1.110)
Animist/Traditional		1.120 (0.859–1.460)		0.972 (0.747–1.264)
No religion		1.185 (1.007–1.394)*		0.972 (0.814–1.161)
Other		0.733 (0.346–1.550)		0.652 (0.301–1.410)
Educational status				
no education		Ref		Ref
primary		0.947 (0.862–1.040)		0.997 (0.908–1.095)
secondary		1.115 (0.994–1.252)		1.080 (0.960–1.215)
higher		2.147 (1.657–2.781)***		1.879 (1.442–2.447)***
Wealth index				
poorest		Ref		Ref
poorer		1.074 (0.975–1.183)		1.073 (0.973–1.182)
middle		1.135 (1.024–1.258)*		1.134 (1.021–1.258)*
richer		1.315 (1.173–1.475)***		1.383 (1.229–1.557)***
richest		1.425 (1.235–1.644)***		1.705 (1.466–1.982)***
Maternal Occupation				
not working		Ref		Ref
Professional/Technical		0.853 (0.723–1.007)		0.971 (0.822–1.147)
Sales		1.215 (1.067–1.384)**		1.095 (0.960–1.250)
Agricultural		0.672 (0.619–0.729)***		0.826 (0.760–0.897)***
Household/Domestic		0.897 (0.790–1.018)		1.002 (0.881–1.141)
Other Services		1.068 (0.653–1.747)		1.231 (0.755–2.007)
Manual Worker		0.836 (0.737–0.949)**		0.947 (0.832–1.077)
Frequency of reading newspaper				
not at all		Ref		Ref
at least once a week		1.034 (0.923–1.158)		1.053 (0.939–1.180)
almost every day		0.981 (0.843–1.142)		1.070 (0.918–1.246)
Frequency of listening to radio				
not at all		Ref		Ref
at least once a week		1.039 (0.953–1.134)		1.005 (0.921–1.096)
almost every day		0.957 (0.882–1.038)		0.992 (0.915–1.077)
Frequency of watching television				
not at all		Ref		Ref
at least once a week		1.461 (1.308–1.631)***		1.198 (1.070–1.340)**
almost every day		1.788 (1.606–1.992)***		1.319 (1.179–1.475)***
Number of ANC visits				
Below 4		Ref		Ref
4–7 visits		1.473 (1.375–1.577)***		1.354 (1.264–1.450)***
8 or more		2.147 (1.824–2.528)***		1.456 (1.230–1.724)***
Time of ANC registration				
1–3 months		Ref		Ref
4–6 months		0.767 (0.714–0.825)***		0.821 (0.763–0.882)***
7+ months		0.605 (0.529–0.693)***		0.621 (0.542–0.712)***
Person providing assistance during ANC				
Doctor		Ref		Ref
Nurse/Midwife		0.812 (0.744–0.887)***		0.773 (0.708–0.845)***
Other health worker		0.792 (0.644–0.975)*		0.619 (0.503–0.762)***
TBA/Other		0.550 (0.427–0.707)***		0.584 (0.454–0.750)***
Place of residence				
urban			Ref	Ref
rural			0.330 (0.297–0.366)***	0.675 (0.593–0.768)***
Distance to health facility a problem				
not a big problem			Ref	Ref
big problem			0.874 (0.820–0.930)***	0.948 (0.886–1.014)
Country				
South Africa (Ref)			Ref	Ref
Angola			0.102 (0.070–0.147)***	1.327 (1.099–1.602)**
Congo DR			0.031 (0.021–0.045)***	0.457 (0.382–0.548)***
Lesotho			0.846 (0.553–1.294)	7.193 (5.432–9.525)***
Malawi			0.031 (0.021–0.045)***	0.343 (0.285–0.411)***
Mozambique			0.076 (0.053–0.111)***	1 (omitted)
Tanzania			0.126 (0.087–0.184)***	1 (empty)

*p ≤ 0.05, **p-value < 0.001 and *** *p*-value < 0.0001.

The findings of the multilevel logistic regression analysis are detailed in Table 4. The null model (Model I) demonstrated significant clustering in the quality of antenatal care (ANC) at the community level. The intraclass correlation coefficient (ICC) was calculated to be 0.46, indicating that 46% of the total variation in the likelihood of receiving quality ANC was attributable to unobserved community-level factors, while the remaining 54% was due to individual-level differences among women. The median odds ratio (MOR) of 5.2 further quantified this substantial cluster effect, suggesting that if a woman were to relocate from one community to another with a higher probability of quality ANC, her odds of receiving quality care would, on average, increase by a factor of 5.2, solely due to the change in community.

**Table 4:** Random effect model results.

Random effect Model	Model I	Model II	Model III	Model IV
Community-level Variation (95% CI)	2.28 (2.10–2.48)	1.09 (0.98–1.21)	1.06 (0.96–1.17)	0.82 (0.73–0.92)
ICC	0.46	0.3	0.28	0.24
LR Test (*χ*^2^)	6,217.71	2,210.29	2,714.39	1,669.03
(*p*-value)	*p* < 0.0001	*p* < 0.0001	*P* < 0.0001	*P* < 0.0001
Wald chi-square	Ref	1,373.13***	1,979.04***	2,144.01***
MOR	5.2	2.8	2.76	2.42
PCV	Ref	0.52	0.53	0.64
Model fitness				
Log-likelihood	−19,619.7	−15,443.67	−18,209.01	−14,991.78
AIC	39,245.4	30,977.33	36,440.01	30,085.56
BIC	39,270.63	31,347.34	36,532.32	30,504.9

Model I = null (no covariates). Model II = individual-level covariates (SWPER domains [ref. = low empowerment], age [ref. = 15–19y], religion [ref. = Christianity], education [ref. = no education], wealth [ref. = poorest], occupation [ref. = not working], newspaper/radio/television exposure [ref. = not at all], ANC visits [ref. = below 4], ANC registration timing [ref. = 1–3 months], ANC provider [ref. = doctor]). Model III = community-level covariates (place of residence [ref. = urban], distance to health facility [ref. = not a big problem], sex of household head [ref. = male], country [ref. = South Africa]). Model IV = Models II + III combined. Full category listings and reference groups for every covariate are given in Table 4.

*** *p*-value < 0.0001.

The introduction of individual-level factors, including the SWPER empowerment domains alongside socio-demographic characteristics, media exposure, and ANC service factors, in Model II explained a substantial portion of this community-level variation. The community-level variance dropped from 2.28 to 1.09, corresponding to a 52% proportional change in variance (PCV). The ICC decreased to 0.30 and the MOR fell to 2.8, indicating that individual-level factors, including but not limited to women's empowerment, account for a major share of the geographical disparities in quality ANC. Model III, which incorporated community-level factors (place of residence, distance to health facility, sex of household head, and country), further improved the model fit. The community-level variance was slightly reduced to 1.06 (PCV = 53%), and the model's explanatory power increased, as evidenced by the highly significant Wald chi-square (*χ*^2^ = 1,979.04, *p* < 0.001).

The full model (Model IV), which included all individual, household, health system, and country-level factors, demonstrated the best fit and explained the greatest share of community-level variation. The community-level variance was reduced to 0.82, representing a 64% reduction from the null model (PCV = 0.64). The ICC (0.24) and MOR (2.42), while still significant, were substantially lower than in the null model. This indicates that the final model's covariates successfully explained nearly two-thirds of the initial unexplained differences between communities. The persistent, though diminished, significant community-level variance (*p* < 0.0001) suggests that other unmeasured contextual factors, such as specific health facility characteristics or regional policies, still play a role.

All models showed a significant improvement over the null model, as confirmed by the likelihood ratio (LR) tests (all *p* < 0.0001). The model fitness statistics consistently improved from Model I to Model IV, with the Akaike Information Criterion (AIC) and Bayesian Information Criterion (BIC) decreasing from 39,245.4 and 39,270.63 (Model I) to 30,085.56 and 30,504.9 (Model IV), respectively. This confirms that the sequential inclusion of predictor variables led to a better-fitting and more parsimonious model.

## Discussion

In this multilevel analysis, we attempted to uncover if empowered women, measured by SWPER index, were more likely to receive quality ANC across seven Southern African countries using DHSs. The findings revealed that less than half of women in the study received quality ANC, with significant variation observed between countries. The quality of ANC ranged from 92.1% in South Africa to 27.4% in Malawi. The study findings also showed a significant variation in women's empowerment levels. South Africa and Lesotho consistently showed high levels of women empowerment across all domains. Attitude to violence and social independence were significant predictors of higher odds of receiving quality ANC after adjusting for a covariates. The analysis also confirmed the critical roles of socio-economic status, media exposure, health services and geographic contexts in the uptake of quality ANC. The multilevel model revealed that nearly half of the variation in quality ANC was attributable to unobserved community-level factors, a substantial portion of which was explained by individual-level factors, including but not limited to differences in women's empowerment.

The association between women's empowerment and quality ANC is supported in this study's findings. Specifically, women with high social independence and those who rejected attitudes justifying gender-based violence had higher odds of receiving quality ANC. This finding corresponds with studies form SSA that have also demonstrated a strong link between women empowerment and quality ANC (17, 18). Also in Malawi, studies have confirmed the intrinsic importance of women empowerment in assessing quality maternal healthcare services including ANC services (32, 33). Our findings suggest that women empowerment may be associated with a greater capacity to seek and receive comprehensive healthcare. Empowered women, who have greater freedom of movement and social autonomy and who uphold rights against violence, may be better positioned to seek and receive comprehensive healthcare. It is also important to note that the significant associations observed between women empowerment and quality ANC could be attributed to the large data employed in the study. The interpretation of this finding could therefore benefit from contextual interpretation, rather than based on an absolute statistical significance.

Empowered women who have greater freedom of movement and autonomy may not require to seek permission to decide to go the clinic as soon as she suspects she is pregnant. This allows her to register in the first trimester, which is critical for baseline tests and starting preventive treatments like iron and folic acid. Additionally, high social independence would enable the woman to repeatedly leave home to visit the ANC clinic to make up the recommended ANC 4–8 visits without requiring permission from anyone. The ability to move freely allows women to interact with other women in the community, community health workers, and other social networks. This sort of informal information sharing is a powerful tool for learning about the importance of ANC. These women are likely to share what to expect during ANC visits and which facilities provide good care.

In the same vein, women who believe that violence is not justified for actions like arguing or going out without permission is likely to be in a relationship with a partner who is less controlling and supports her autonomy. In such instances, the women are less likely to face opposition for making independent health decisions. She is also more likely to be supported financially and emotionally by her partner in seeking timely and frequent ANC, which is key component of receiving quality ANC. These findings are consistent with the wider empowerment and healthcare-seeking literature, rather than causal mechanisms established by this cross-sectional study (34–36).

Decision-making among women still remains a greater concern across most communities in SSA including Southern Africa and its effect in accessing healthcare (34–36). Women largely depends on their husbands or household heads decisions in seeking healthcare, including attending ANC. Women with low social decision making power has little to no control over the household financial resources. Therefore, if the decision-maker does not prioritize ANC or sees it as an unnecessary expense, funds may not be made available for her to seek ANC services.

The stark cross-national variation in empowerment shows that country-specific social, cultural, and legal contexts are fundamental in shaping these domains and how they interact with maternal healthcare seeking behaviours. For instance in South Africa, which demonstrated very high levels of women empowerment, as well as, high quality ANC have created and integrated its National Policy Framework for Women's Empowerment and Gender Equality into key agencies including the healthcare agency (37). Other countries like Tanzania has made efforts to improve quality ANC, this has however been impacted by contextual challenges like economic factors faced by these women in accessing ANC (38, 39). The Coalition for the Empowerment of Women and Girls (CEWAG) in Malawi and other organizations in the country have realized the importance of women empowerment and are taking pragmatic steps to improving women empowerment in the country and increasing their access to optimum maternal healthcare services (33, 40).

Beyond empowerment, this study reaffirms the multifaceted determinants of quality ANC. Socio-economic and geographic factors showed women with higher education, from the richest wealth quintiles, and residing in urban areas had significantly higher odds of receiving quality care. This reflects entrenched inequities in access to resources and health infrastructure, consistent with the literature on ANC in low-resource settings (41, 42). Increasing wealth index association with increased odds of quality ANC suggests that women within the high gradient are able to afford the cost and autonomy of seeking timely and frequent ANC services. Also, higher educational women are more likely to have a high health literacy (43, 44). They are therefore more likely to be well informed on the importance and services available during ANC. The lower odds of receiving quality ANC among rural mothers could be attributed to the long standing health inequalities within urban-rural geographic divides across SSA (36, 45, 46). It is still remains critical for healthcare managers and governments to prioritize ANC service provision in rural areas across Southern Africa.

Media exposure, particularly daily television viewing, emerged as a powerful correlate, highlighting the role of information dissemination in shaping health behaviors. This finding corresponds with an earlier study in SSA involving 13 countries (17). The pervasive nature of television and its ability to reach diverse audiences makes it a potent medium for conveying health-related messages. This is done through either news reports, public service announcements or health-related programs. This exposure, as evident in this study, can significantly influence viewers' awareness, knowledge, and attitude towards various health issues including the importance of ANC.

The most potent predictors of quality ANC were health service factors. A very strong relationship was established between quality ANC and the month at ANC registration, the number of ANC visits and the person providing ANC services. A very significant increased odds for quality ANC was observed among mothers who registered very early (between 1 and 3 months), and mothers who made more than 4 ANC visits. These findings imply that women who initiate care early and attends frequently is far more likely to receive all essential components of ANC. This finding is corroborated by earlier studies in similar LMICs (47, 48). This reiterate the importance of implementing WHO's guidelines on ANC for a positive pregnancy experience (49).

The results also showed that the Mozambique coefficient was omitted because of exact collinearity, while the Tanzania estimate became unstable with a substantially inflated standard error. This instability arose from structural differences in questionnaire content across the pooled DHS surveys. Religion was not collected in South Africa and Tanzania, while occupation was collected only in Tanzania. Consequently, the distribution of these covariates across countries created exact or near-exact collinearity when they were included alongside country indicators in the fully adjusted model. This represents a limitation of the pooled-data specification rather than a data or coding error. The Mozambique and Tanzania coefficients in Model IV should therefore be interpreted cautiously.

Southern African countries should recognize that higher levels of social independence and rejection of gender-based violence significantly improve uptake of quality ANC. Therefore, their health policies should prioritize integrating women's empowerment into maternal health strategies. Governments in low-performing countries such as Malawi and Congo DR should draw lessons from South Africa and Lesotho, where strong empowerment frameworks and gender equality policies have translated into higher-quality ANC coverage. It is also essential to enhance health system capacity by promoting early ANC registration and adhering to the WHO-recommended ANC visits. For practice, community-level interventions that support women's autonomy and reduce financial and geographic barriers can play a transformative role. Further research should explore unmeasured contextual factors, such as health facility quality and local policy implementation. Also, country-specific studies should identify and evaluate scalable, context-appropriate strategies for improving both women's empowerment and maternal health outcomes across the region.

## Strength and limitations

This study leverages large, nationally representative DHS datasets across seven Southern African countries. This provides a robust evidence on the relationship between women's empowerment and quality ANC. The use of the validated SWPER Global Index strengthens the reliability of empowerment measurement. Additionally, the multilevel analytical approach allowed disentangling of individual and community-level factors that influence ANC quality. Moreover, cross-country comparisons highlight critical contextual differences that can guide tailored policy interventions.

However, the study has some limitations. The cross-sectional design precludes causal inference, and self-reported data may be subject to recall or social desirability bias. The operational definition of quality ANC was restricted to four components due to data limitations. This potentially underestimated comprehensive ANC quality. Finally, unmeasured contextual factors, such as health facility infrastructure or provider competence, were not captured in the DHS datasets but may have influenced the observed associations.

## Conclusion

This study demonstrates that women's empowerment, particularly social independence and rejection of gender-based violence under the attitude to violence domain, significantly improves the uptake of quality antenatal care in Southern Africa. However, large inequities persist across socio-economic, geographic, and service-related dimensions, with women in rural areas, those with low education, and those from poorer households consistently disadvantaged. The strong influence of service-related factors, such as early ANC registration, frequency of visits, and type of provider, underscores the need to strengthen health systems alongside promoting gender equality. Policies integrating women's empowerment strategies with maternal health programs, as seen in South Africa and Lesotho, offer promising pathways for other countries such as Malawi and the Democratic Republic of Congo, where uptake remains low. To accelerate progress toward SDG maternal health targets, Southern African governments must prioritize both empowerment and structural health system reforms.

## Data Availability

The datasets presented in this study can be found in online repositories. The names of the repository/repositories and accession number(s) can be found below: The DHS Program (https://www.dhsprogram.com/).
