# Supplementary material for: Leveraging women empowerment for better maternal health: using the SWPER index to identify key drivers of quality antenatal care in Southern Africa
Source: Front Glob Womens Health. 2026 Oct 2;7:1886495. doi: 10.3389/fgwh.2026.1886495 (PMC13672230; doi:10.3389/fgwh.2026.1886495)
Supplement: Supplementary file 1 [file Table1.docx]

Supplementary Table 1: Countries, study population, and sample size included in the study

| **Country** | **Year** | **Study population** | **Unweighted Final sample** | **Weighted sample** |
| --- | --- | --- | --- | --- |
| Angola | 2015 - 16 | 14379 | 5,611 | 5548 |
| Congo DR | 2023 - 24 | 27583 | 10,035 | 8965 |
| Lesotho | 2023 - 24 | 6413 | 1,015 | 984 |
| Malawi | 2015 - 16 | 24562 | 10,767 | 10873 |
| Mozambique | 2022 - 23 | 13183 | 3,685 | 3759 |
| South Africa | 2016 | 8514 | 1,130 | 1241 |
| Tanzania | 2022 | 15254 | 4,428 | 4492 |
| **Total** |  | **109,888** | **36,671** | **35,862** |
