# Supplementary material for: Leveraging women empowerment for better maternal health: using the SWPER index to identify key drivers of quality antenatal care in Southern Africa
Source: Front Glob Womens Health. 2026 Oct 2;7:1886495. doi: 10.3389/fgwh.2026.1886495 (PMC13672230; doi:10.3389/fgwh.2026.1886495)
Supplement: Supplementary file 2 [file Table2.docx]

Supplementary Table 2: Generalized Variance Inflation Factor

| **Variable** | **VIF** | **1/VIF** |
| --- | --- | --- |
| wealth index | 1.92 | 0.52 |
| Frequency of watching television | 1.77 | 0.57 |
| Place of residence | 1.74 | 0.57 |
| Maternal educational level | 1.67 | 0.60 |
| Country | 1.62 | 0.62 |
| Social independence | 1.5 | 0.67 |
| Number of ANC visits | 1.25 | 0.80 |
| Marital status | 1.25 | 0.80 |
| Frequency of reading newspaper | 1.25 | 0.80 |
| Religion | 1.24 | 0.81 |
| Month at ANC registration | 1.23 | 0.82 |
| Frequency of listening to radio | 1.22 | 0.82 |
| Attitude to violence | 1.19 | 0.84 |
| Maternal age | 1.16 | 0.86 |
| Distance to health facility | 1.09 | 0.92 |
| Decision making | 1.08 | 0.92 |
| Maternal occupation | 1.06 | 0.94 |
| Person providing ANC | 1.02 | 0.98 |
| Sex of household head | 1.01 | 0.99 |
| **Mean VIF** | **1.33** |  |
| **Generalized VIF (GVIF)** | **1.31** |  |
