# Supplementary material for: Leveraging women empowerment for better maternal health: using the SWPER index to identify key drivers of quality antenatal care in Southern Africa
Source: Front Glob Womens Health. 2026 Oct 2;7:1886495. doi: 10.3389/fgwh.2026.1886495 (PMC13672230; doi:10.3389/fgwh.2026.1886495)
Supplement: Supplementary file 3 [file Table3.docx]

Supplementary Table 3: Baseline characteristics of the study sample, n = 35862.

| **Socio-demographic Characteristics** | **Weighted Frequency (n = 35862)** | **Percent (%)** |  |
| --- | --- | --- | --- |
|  |  |  |  |
| **Age group of women** | |  |  |
| 15-19 | 2,625 | 7.3 |  |
| 20-24 | 9,411 | 26.2 |  |
| 25-29 | 8,842 | 24.7 |  |
| 30-34 | 7,086 | 19.8 |  |
| 35-39 | 4,933 | 13.8 |  |
| 40-44 | 2,297 | 6.4 |  |
| 45-49 | 668 | 1.9 |  |
|  |  |  |  |
| **Religion** |  |  |  |
| Christianity | 25,533 | 84.8 |  |
| Islam | 2,541 | 8.4 |  |
| Animist/Traditional | 588 | 2.0 |  |
| No religion | 1,422 | 4.7 |  |
| Other | 44 | 0.2 |  |
|  |  |  |  |
| **Educational status** | |  |  |
| no education | 6,455 | 18.0 |  |
| primary | 16,523 | 46.1 |  |
| secondary | 11,607 | 32.4 |  |
| higher | 1,277 | 3.6 |  |
|  |  |  |  |
| **Wealth index** |  |  |  |
| poorest | 7,804 | 21.8 |  |
| poorer | 7,889 | 22.0 |  |
| middle | 7,036 | 19.6 |  |
| richer | 6,844 | 19.1 |  |
| richest | 6,289 | 17.5 |  |
|  |  |  |  |
| **Place of residence** |  |  |  |
| urban | 11,353 | 31.7 |  |
| rural | 24,509 | 68.3 |  |
|  |  |  |  |
| **Maternal Occupation** | |  |  |
| not working | 12,803 | 35.7 |  |
| Professional/Technical | 1,645 | 4.6 |  |
| Sales | 2,570 | 7.2 |  |
| Agricultural | 12,267 | 34.2 |  |
| Household/Domestic | 2,644 | 7.4 |  |
| Other services | 249 | 0.7 |  |
| Manual worker | 3,497 | 9.8 |  |
| Other/Missing | 185 | 0.5 |  |
|  |  |  |  |
| **Frequency of reading newspaper** | |  |  |
| not at all | 29,917 | 83.4 |  |
| at least once a week | 3,703 | 10.3 |  |
| almost every day | 2,242 | 6.3 |  |
|  |  |  |  |
| **Frequency of listening to radio** | |  |  |
| not at all | 19,534 | 54.5 |  |
| at least once a week | 6,922 | 19.3 |  |
| almost every day | 9,407 | 26.2 |  |
|  |  |  |  |
| **Frequency of watching television** | |  |  |
| not at all | 24,361 | 67.9 |  |
| at least once a week | 3,706 | 10.3 |  |
| almost every day | 7,795 | 21.7 |  |
|  |  |  |  |
| **Number of ANC visits** | |  |  |
| No ANC | 2,982 | 8.3 |  |
| Below 4 | 12,428 | 34.7 |  |
| 4 - 7 visits | 18,636 | 52.1 |  |
| 8 or more | 1,742 | 4.9 |  |
|  |  |  |  |
| **Time of ANC registration** | |  |  |
| No ANC | 2,982 | 8.3 |  |
| 1-3 months | 10,503 | 29.4 |  |
| 4-6 months | 19,710 | 55.1 |  |
| 7+ months | 2,593 | 7.2 |  |
|  |  |  |  |
| **Person providing assistance during ANC** | |  |  |
| Doctor | 5,766 | 16.2 |  |
| Nurse/Midwife | 21,427 | 60.2 |  |
| Other health worker | 4,817 | 13.5 |  |
| TBA/Other | 622 | 1.8 |  |
| No ANC | 2,939 | 8.3 |  |
|  |  |  |  |
| **Distance to health facility a problem** | |  |  |
| not a big problem | 19,106 | 54.3 |  |
| big problem | 16,104 | 45.7 |  |
|  |  |  |  |
| **Sex of household head** | |  |  |
| male | 30,017 | 83.7 |  |
| female | 5,845 | 16.3 |  |
|  |  |  |  |
| **Country** |  |  |  |
| Angola | 5548 | 15.5 |  |
| Congo DR | 8965 | 25 |  |
| Lesotho | 984 | 2.7 |  |
| Malawi | 10873 | 30.3 |  |
| Mozambiq | 3759 | 10.5 |  |
| South Af | 1241 | 3.5 |  |
| Tanzania | 4492 | 12.5 |  |
